# A Novel Finding of Retinal Neovascularization Secondary to Chronic Central Serous Chorioretinopathy

**DOI:** 10.7759/cureus.88806

**Published:** 2025-07-26

**Authors:** Chathuranka De Silva, Ajay Kotagiri

**Affiliations:** 1 Ophthalmology, Royal Victoria Infirmary, Newcastle upon Tyne, GBR; 2 Ophthalmology, Sunderland Eye Infirmary, Sunderland, GBR

**Keywords:** anti-vegf treatment, central serous chorioretinopathy, complication, pan-retinal photocoagulation laser, retinal neovascularization, retina

## Abstract

We report a rare case of a 59-year-old male with bilateral chronic multifocal central serous chorioretinopathy (CSCR) who developed retinal neovascularization (RNV). This patient had been under review for CSCR for several years before developing a new frond of RNV in his right eye. Comprehensive investigations, including fundus fluorescein angiography, were performed to exclude alternative causes such as diabetic retinopathy, vascular occlusion, or retinal vasculitis. He was treated with three intravitreal injections of bevacizumab and received sectoral panretinal photocoagulation laser with good response and regression of the vessel appearance.

## Introduction

Central serous chorioretinopathy (CSCR) is the fourth most common retinopathy after age-related macular degeneration, diabetic retinopathy, and branch retinal vein occlusion [1]. CSCR is a retinal disease characterized by serous detachment of the neurosensory retina, most commonly involving the macula. It is frequently associated with pigment epithelial detachments (PEDs), dysfunction of the retinal pigment epithelium (RPE), choroidal thickening, increased choroidal vascular permeability, and venous outflow congestion [2]. Clinically, patients typically present with symptoms such as blurred vision, central scotoma, micropsia, or metamorphopsia. Although historically considered a primary disorder of the retina, more recent evidence has highlighted the integral roles of the choroid, RPE, and sclera in its pathogenesis. As a result, CSCR is now widely recognized as a manifestation within the broader pachychoroid disease spectrum [3].

Well-documented complications of CSCR include secondary choroidal neovascularization; however, complications such as retinal neovascularization (RNV) are exceedingly rare, with only a few reports in the literature. We report the case of a 59-year-old male with a longstanding history of CSCR, who developed RNV. The patient had been under regular ophthalmic surveillance for more than a decade due to persistent subretinal fluid and progressive outer retinal atrophy. His case is notable for the absence of typical systemic risk factors and the emergence of retinal neovascularization in an eye with chronic disease-related changes.

## Case presentation

A 59-year-old male patient was referred initially 11 years prior for metamorphopsia in the right eye and was noted to have PED with subretinal fluid compatible with CSCR. The patient had no prior history of corticosteroid use, either systemic, inhaled, or topical. His medical history was otherwise unremarkable with no known systemic diseases, including diabetes mellitus, hypertension, or cardiovascular disease. He denied tobacco use and had no family history of retinal vascular or degenerative conditions.

His CSCR was noted to be multifocal, and, eventually, his fellow eye became affected. He had undergone multiple therapeutic interventions, including a course of oral eplerenone, which produced only a transient reduction in subretinal fluid. In the right eye, he had foveal involving chronic subretinal fluid tracking inferiorly with outer retinal atrophy and chronic visual loss. In the left eye, he had extrafoveal areas of chronic subretinal fluid where focal laser had been performed to address localized areas of active leakage, with success and preserved vision.

In one review, around 11 years after his initial presentation, he reported no acute visual changes. Visual acuity remained asymmetric, with 50 ETDRS letters (Snellen 6/30) in the right eye and 90 ETDRS letters (Snellen 6/5) in the left eye. Intraocular pressures were 12 mmHg in both eyes. Anterior segment examination was unremarkable with no signs of inflammation, iris neovascularization, or lens opacities.

Posterior segment examination revealed subtle RPE changes and retinal elevation in both maculae consistent with previous findings. In the right eye, however, a new frond of RNV was identified along the inferotemporal arcade. There was no evidence of vitreous cells or haze to suggest intraocular inflammation or vitreous hemorrhage. His optic nerves appeared healthy, and the peripheral retina and vessels looked healthy with no signs of diabetic retinopathy, retinal vein occlusion, or retinal vasculitis.

**Figure 1:**
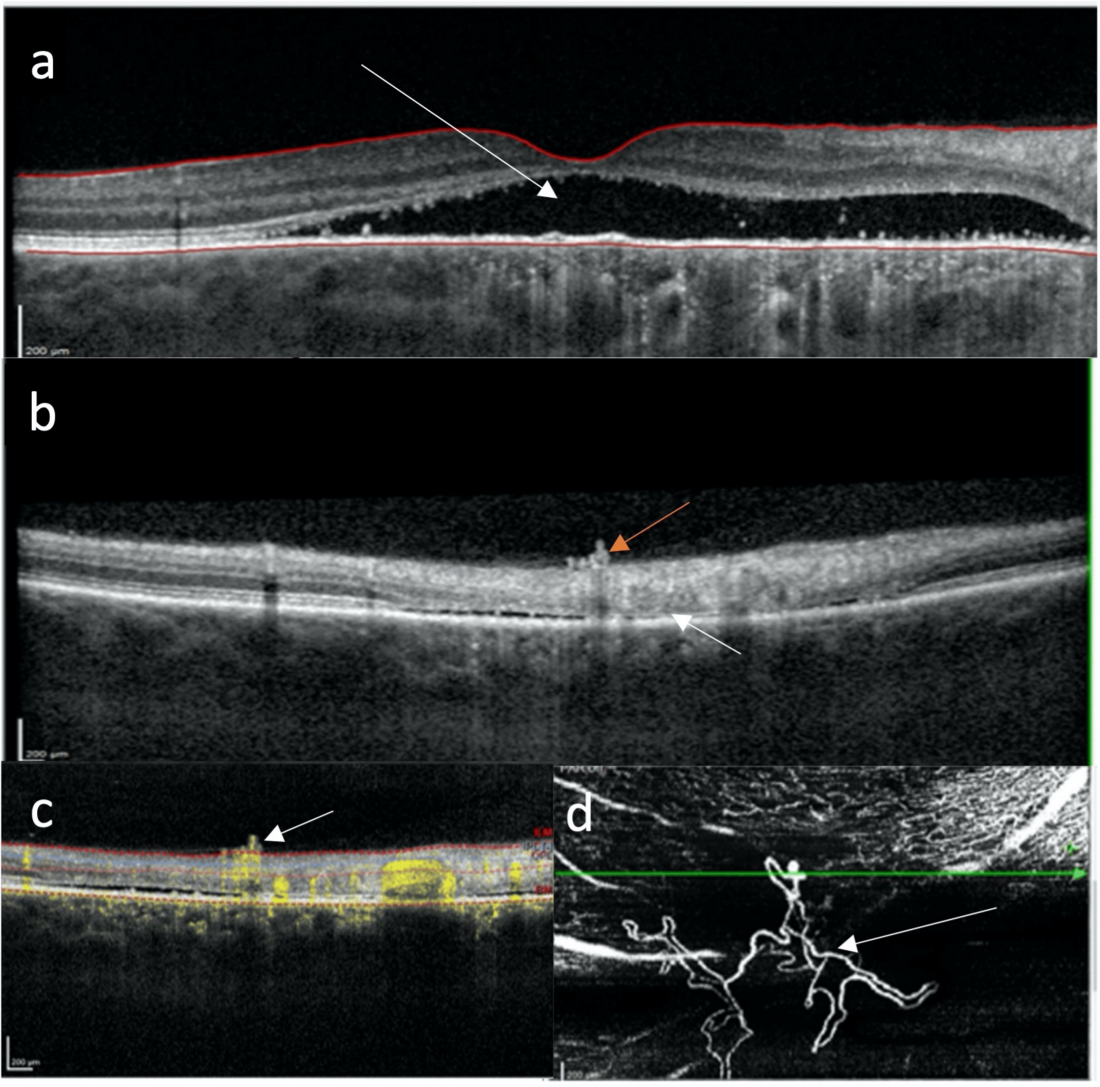
(a) Heidelberg spectral-domain optical coherence tomography (OCT) showing right eye persisting subfoveal subretinal fluid (arrow). (b) Heidelberg spectral-domain OCT showing right eye outer retinal atrophy (white arrow) and overlying retinal neovascularization (orange arrow). (c) Heidelberg OCT-angiography confirming retinal neovasculariazation with a high signal flow (arrow). (d) Heidelberg OCT-angiography confirming a retinal neovascular frond in the superficial capillary plexus layer (arrow).

Spectral-domain optical coherence tomography (OCT) confirmed persistent subretinal fluid in both eyes, which was subfoveal in the right eye (Figure 1). OCT also demonstrated in the right eye an inferior area of photoreceptor and RPE loss where the overlying RNV was present (Figure 1). OCT angiography (OCT-A) further confirmed the RNV to have high signal flow and to be located at the level of the superficial vascular complex (Figure 1).

**Figure 2:**
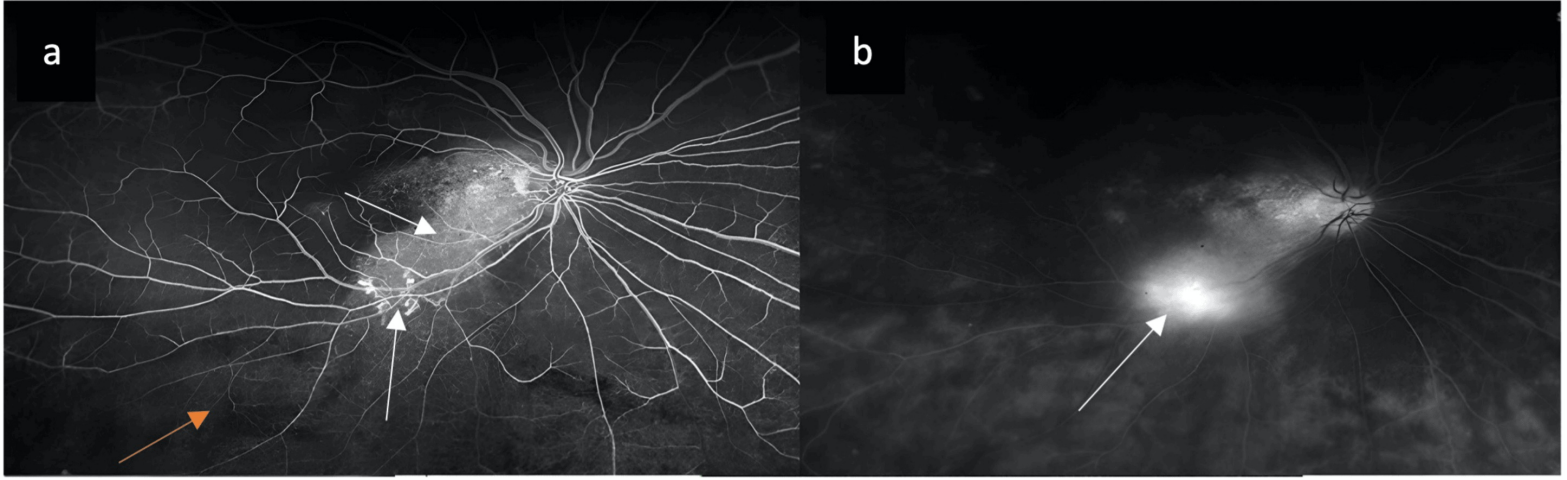
(a) Fundus fluorescein angiography (FFA) in arteriovenous phase showing hyperfluorescent areas correlating with retinal pigment epithelium defects and retinal neovascularization (white arrows), hypofluorescent areas inferiorly correlating with areas of non-perfusion (orange arrow). (b) FFA in late phase confirming active leakage from retinal neovascularization (arrow).

Fundus fluorescein angiography (FFA) revealed normal choroidal filling time (12 seconds). It also revealed bilateral window defects, consistent with chronic RPE disruption. Importantly, there was focal leakage corresponding to the right eye RNV, confirming its activity (Figure 2). There were also inferior areas of peripheral non-perfusion and capillary dropout in the right eye, suggestive of possible peripheral ischemia (Figure 2). There were no areas of peripheral ischemia or new vessels in the left eye.

**Figure 3:**
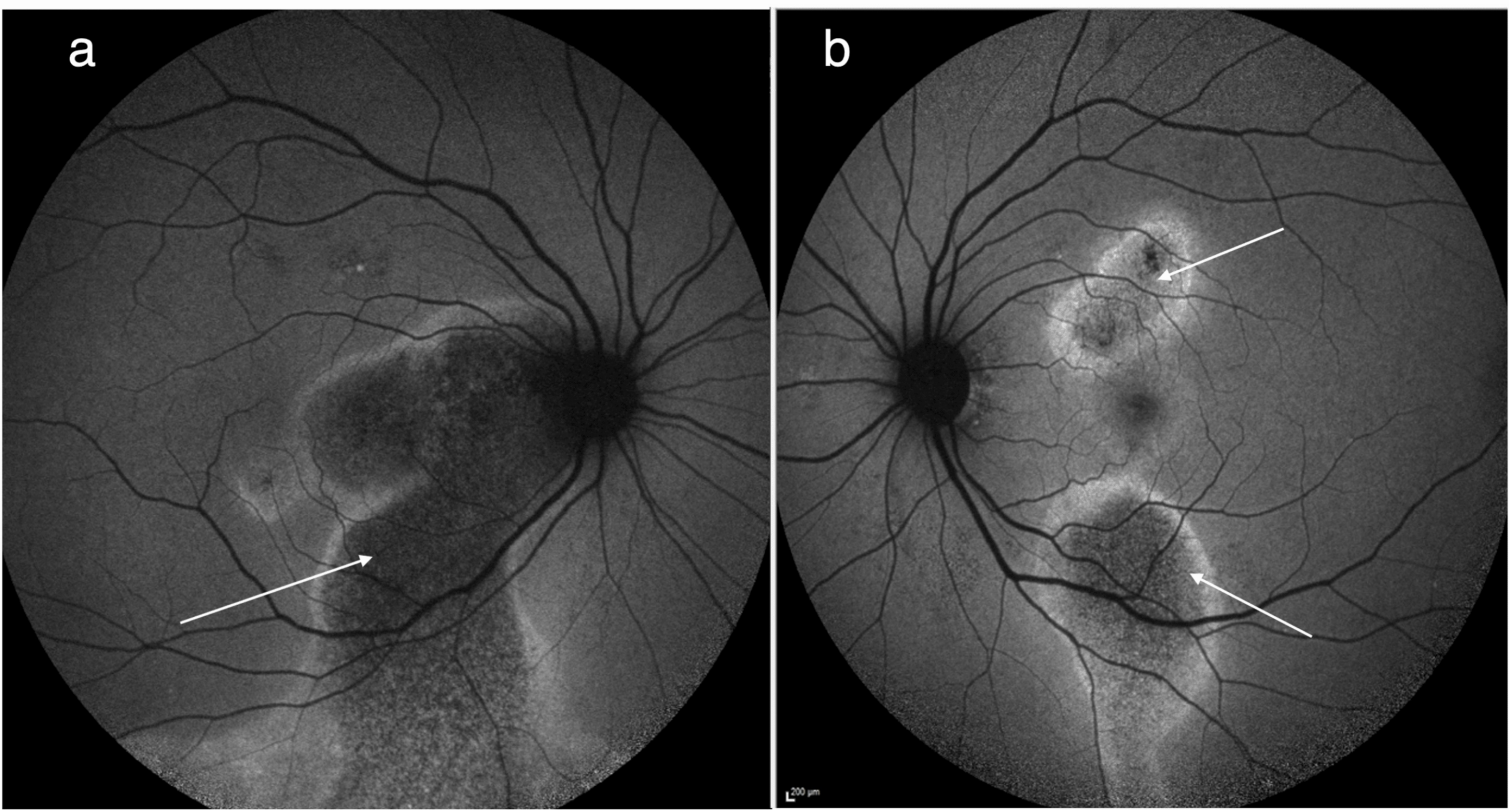
(a) Fundus autofluorescence (FAF) of the right eye showing foveal involving area of hypo autofluorescence surrounded by a halo of increased hyper autofluorescence (arrow). (b) FAF of the left eye showing foveal sparing areas of hypo autofluorescence surrounded by haloes of increased hyper autofluorescence.

Fundus autofluorescence (FAF) imaging provided additional insights into the chronicity of disease. Both maculae exhibited large areas of hypo autofluorescence surrounded by haloes of increased hyper autofluorescence, findings consistent with gravitational tracking of subretinal fluid and pigment epithelial changes typically observed in chronic CSCR (Figure 3).

Given the clinical context and detailed multimodal imaging, there was no evidence to support alternative causes such as diabetic retinopathy, ocular ischemic syndrome, retinal vein occlusion, or retinal vasculitis. We, therefore, attributed the RNV observed in this patient to chronic CSCR, representing a rare but plausible complication of the disease.

**Figure 4:**
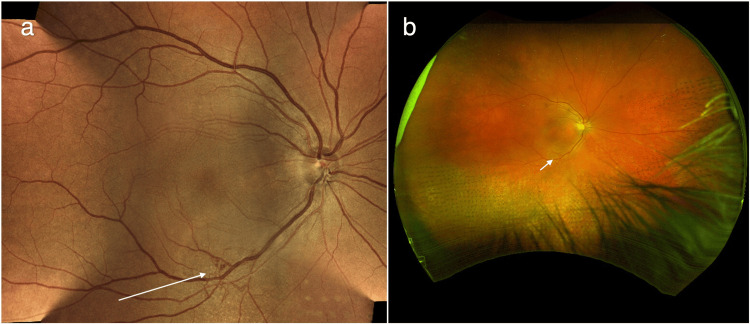
(a) Centervue DRSplus retinal imaging depicting retinal neovascularization (RNV) (arrow). (b) Optos imaging showing regression of RNV (arrow) several months after sectoral panretinal photocoagulation laser treatment.

The patient underwent three intravitreal injections of bevacizumab to the right eye as required, with inferior sectoral panretinal photocoagulation (PRP) laser to the non-perfused inferior areas of the retina on FFA. His most recent images showed regression of the new vessel following his treatment (Figure 4).

## Discussion

RNV is a well-recognized complication of inner retinal ischemic conditions, including retinal vein occlusion, diabetic retinopathy, ocular ischemic syndrome, retinopathy of prematurity, vitreoretinopathies, sickle cell retinopathy, radiation retinopathy, and inflammatory vasculitis [4]. However, the development of RNV in the context of CSCR is exceedingly rare, with only a few reports in the literature [5,6]. The pathophysiological mechanism by which CSCR leads to RNV remains speculative. It is postulated that, like the pathophysiological mechanisms observed in chronic rhegmatogenous retinal detachment, prolonged neurosensory retinal detachment and outer retinal atrophy in chronic CSCR can lead to hypoxia and localized inner retinal ischemia. This may then induce cellular stress and trigger the release of pro-inflammatory cytokines, leading to a vasculitis-like response [7]. Such inflammation may contribute to retinal vascular occlusions, peripheral capillary dropout, and the formation of areas of non-perfusion [8]. Persistent ischemia in these zones is then believed to stimulate the upregulation of angiogenic mediators, particularly vascular endothelial growth factor (VEGF), thereby promoting abnormal retinal neovascularization.

When RNV is identified, it is essential to exclude the more common etiologies such as diabetic retinopathy, retinal vascular occlusions, ocular ischemic syndrome, and vasculitis. In our case, comprehensive clinical evaluation and multimodal imaging successfully ruled out these differential diagnoses, with all findings aligning with a diagnosis of chronic CSCR.

Managing neovascular complications in CSCR presents a significant clinical challenge. While laser photocoagulation remains the standard approach for targeting ischemic areas, its effectiveness is often restricted by the extent and location of the neurosensory detachment. In cases where laser therapy may be insufficient, intravitreal anti-VEGF injections offer a viable alternative, as demonstrated in our patient. We started with anti-VEGF injections to shrink the vessel quickly and then went on to do retinal laser treatment for more long-term effects. Ongoing monitoring is crucial to assess the response to treatment, with a view to further adjunctive anti-VEGF injections or laser therapy if felt necessary.

## Conclusions

Our case underscores a rare and atypical manifestation of RNV in chronic CSCR, adding to the spectrum of its clinical presentations and diagnostic complexity. Moreover, it highlights the potential for successful management of such cases using an anti-VEGF agent along with sectoral panretinal photocoagulation laser treatment with long-term follow-up.
